# Revealing the mechanism of passive transport in lipid bilayers via phonon-mediated nanometre-scale density fluctuations

**DOI:** 10.1038/ncomms11575

**Published:** 2016-05-12

**Authors:** Mikhail Zhernenkov, Dima Bolmatov, Dmitry Soloviov, Kirill Zhernenkov, Boris P. Toperverg, Alessandro Cunsolo, Alexey Bosak, Yong Q. Cai

**Affiliations:** 1 National Synchrotron Light Source II, Brookhaven National Laboratory, Upton, New York 11973, USA; 2 Frank Laboratory of Neutron Physics, Joint Institute for Nuclear Research, Dubna 141980, Russia; 3 Moscow Institute of Physics and Technology, Dolgoprudny 141700, Russia; 4 Institut Nanosciences et Cryogénie, Commissariat à l'Energie Atomique, Grenoble 38054, France; 5 Petersburg Nuclear Physics Institute, Gatchina 188300, Russia; 6 Institut Laue Langevin, 6, rue Jules Horowitz, Grenoble 38042, France; 7 European Synchrotron Radiation Facility, Grenoble 38000, France

## Abstract

The passive transport of molecules through a cell membrane relies on thermal motions of the lipids. However, the nature of transmembrane transport and the precise mechanism remain elusive and call for a comprehensive study of phonon excitations. Here we report a high resolution inelastic X-ray scattering study of the in-plane phonon excitations in 1,2-dipalmitoyl-*sn*-glycero-3-phosphocholine above and below the main transition temperature. In the gel phase, for the first time, we observe low-frequency transverse modes, which exhibit a phonon gap when the lipid transitions into the fluid phase. We argue that the phonon gap signifies the formation of short-lived nanometre-scale lipid clusters and transient pores, which facilitate the passive molecular transport across the bilayer plane. Our findings suggest that the phononic motion of the hydrocarbon tails provides an effective mechanism of passive transport, and illustrate the importance of the collective dynamics of biomembranes.

## Abstract (web summary)

The molecular transport through bio-membranes of cells heavily relies on the dynamics of lipids, but the related mechanism remains unknown. Here, Zhernenkov *et al*. observe the propagating transverse phonon mode with a finite band gap and suggest its connection to short-lived local lipid clustering.

## Graphical abstract

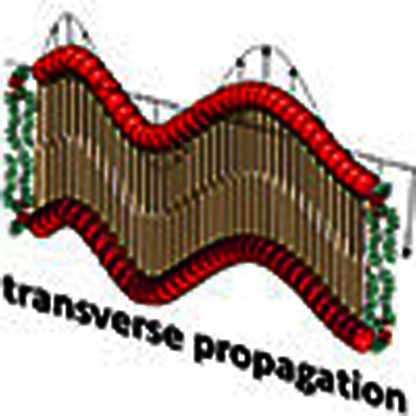

A biological membrane serves as a selective barrier between the internal compartment of the cell and the cell's surroundings. Because it mediates a variety of processes (such as transport of small molecules, permeation of ions, protein budding and trafficking, and signal transduction), its structure and composition are necessarily complex. Biological membranes are dynamic, fluid systems whose constituents are able to move within the membrane's plane^1^. One of the membrane's main constituents is the lipid, which has long been established as the basis for the membrane's structure. Because of their polar head groups and two hydrophobic hydrocarbon tails, lipids spontaneously form bilayers in aqueous environments. Such a lipid bilayer provides both a stable enclosure for the cell contents and the fluidity and mobility required for the cell to carry out its functions, including passive transport of molecules needed to support the biological functions. Examples of such passive transport include proton translocation, the permeation of small polar molecules such as water^2^ or molecules that can dissolve in lipids, like oxygen and carbon dioxide^3^. It has been experimentally shown that many factors, such as solute nature, molecule type and size or membrane thickness define the permeability process^4–6^; however, the exact mechanism of such transport still remains unknown. Different models (such as the solubility-diffusion model, transient pore model or packing defect model, to name a few) that take into account solute hydrophobicity, molecular size and shape, together with membrane fluidity and packing density were proposed to explain the passive transport mechanism. However inconsistencies between the different models and experimental data still persist^7^. Remarkably, all of the proposed models to describe the permeability process through a cell membrane depend on thermally activated vibrations of the lipids in the membrane, which in turn depend on the membrane composition. Although the current understanding of the membrane structure is fairly mature^8^,^9^,^10^,^11^,^12^,^13^,^14^ a robust model for the collective dynamics of lipids is still at its infancy. Therefore, in order to gain a deeper insight into these energy-transfer phenomena across the lipid membrane a systematic and comprehensive study of lipid dynamics is required.

During the past decade, many incoherent dynamic phenomena, such as the ultra-fast rotational and vibrational spectra^15^,^16^,^17^ and diffusion processes in the plane of the membrane^18^,^19^,^20^,^21^,^22^ have gained increasing attention. In addition, extensive efforts have been dedicated to molecular dynamics simulation of collective (coherent) chain dynamics^23^,^24^,^25^,^26^. Still, despite considerable interest and potential wide impact, experimental studies of the fast picosecond collective dynamics in cell membranes have been sporadic. In recent years, there have only been a few experiments utilizing inelastic X-ray (IXS)^25^,^27^,^28^,^29^ and inelastic neutron scattering (INS)^25^,^30^,^31^, where the longitudinal phonon modes and their evolution as a function of temperature were observed. IXS and INS are complementary techniques in studying collective lipid dynamics. INS normally provides superior energy resolution and induce no beam damage on a sample; however, it suffers from kinematic constrains limiting its accessible dynamic range. In INS experiments, the sample must be deuterated in order to remove the large incoherent scattering from the hydrogen atoms in the sample. On the other hand, the IXS scattering cross-section is inherently coherent, so the deuteration of lipids is not required and, apart from the limited energy resolution, the method is virtually free from kinematic limitations. It is important to mention that the selective deuteration of lipid's acyl chains is undesirable since the substitution of hydrogen with deuterium notably changes lipid's physical properties. For example, the main transition temperature of 1,2-dipalmitoyl-*sn*-glycero-3-phosphocholine (DPPC) reduces by ∼5 °C in case of DPPC-d62 deuteration^32^,^33^. It is noteworthy that the dynamics of the fully hydrated DPPC membrane is particularly interesting, since this lipid is a major constituent of the pulmonary surfactant, through which oxygen must penetrate before it can reach the lung tissue^34^. The pulmonary surfactant is a mixture of phospholipids and proteins containing ∼40% DPPC, a powerful surfactant molecule, which reduces the surface tension to nearly zero^35^ facilitating oxygen transport. Therefore, studying the thermally induced ultra-fast vibrations of DPPC is crucial for understanding the oxygen permeation, in particular, and, more generally, the nature of transmembrane small molecule passive transport^7^.

In this work, we report an IXS study of the in-plane phonon excitations in fully hydrated DPPC above and below the main transition temperature (*T*_m_=41 °C). We provide clear evidence for the high-frequency longitudinal phonon modes previously predicted by molecular dynamics simulations in the L_β'_ (gel) phase^23^, and for their apparent softening upon the DPPC lipid transition into the L_α_ (fluid) phase. We also provide compelling evidence for an in-plane transverse acoustic modes that, to the best of our knowledge, have not been reported before in the literature. The explanation of the transverse modes in DPPC lipid is given within the framework of the phonon theory of liquids^36^,^37^,^38^. Ultimately, we discuss and explain the importance of the observed low-frequency phononic band gap in the context of solute diffusion in lipid bilayers, which highlights the crucial role of phonons for the passive transmembrane transport.

## Results

### Experimental details

**Figure 1:**
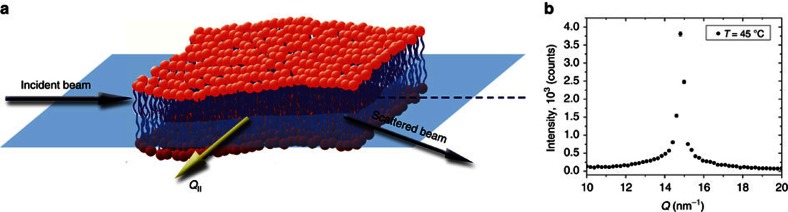
Geometry of IXS experiment and DPPC structure factor. (**a**) In-plane scattering geometry used at the IXS beamline (ID28) at ESRF. The incident and scattered beams remain within the lipid membrane plane and thus the momentum transfer vector *Q* lies parallel to the sample surface. (**b**) S(*Q*,0) as measured showing the chain correlation peak for 45 °C. ESRF, European Synchrotron Radiation Facility.

**Figure 2:**
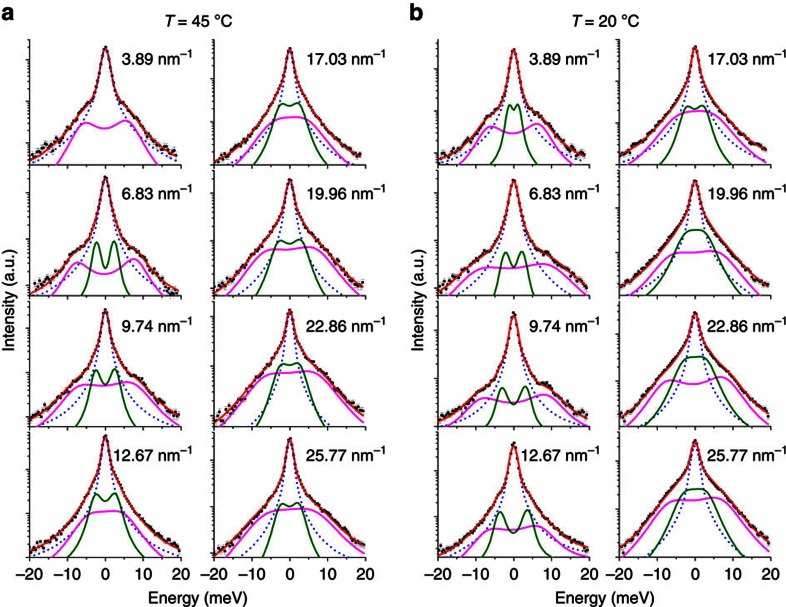
IXS spectra of DPPC. (**a**) IXS spectra measured at *T*=45 °C in the fluid phase and at (**b**) *T*=20 °C in the gel phase. The experimental data (black squares) with error bars signifying 1 s.d. are reported together with the best least squares fit (red solid curves) using a DHO model with two excitations. The DHO models are presented after convolution with the IXS spectrometer RF (denoted by the dashed blue line). Low- and high-frequency DHO are denoted by the green and magenta solid curves, respectively. The corresponding *Q* values are shown in each plot.

The geometry of the IXS experiment is presented in Fig. 1a: the orientation of the scattering vector *Q* is kept within the membrane's plane to ensure that the probed collective dynamics of lipids is preferentially occurring in the lateral plane. As it was shown previously, in an IXS experiment, the major contribution to the measured phonons stems from the collective excitations of lipid tails^25^,^27^,^28^,^30^,^31^. In addition, in such experiments, the peak in the *S*(*Q*) is observed at the position corresponding to the tail-to-tail distance (not head-to-head distance), which can be considered as an independent proof that the major contribution to the measured phonons come from the tails, not the heads groups. The main phase transition temperature of DPPC is 41 °C with the pre-transition to a metastable ripple phase, P_β'_, occurring at 35 °C. Therefore we performed the IXS measurements at 20 and 45 °C at ambient pressure to ensure that the metastable P_β'_ phase was avoided. Selected IXS spectra for different *Q*-values for the measurements performed at 20 and 45 °C are presented in Fig. 2 together with the total fit (red solid curves) and its individual damped harmonic oscillator (DHO) components.

### IXS spectra fitting

**Figure 3:**
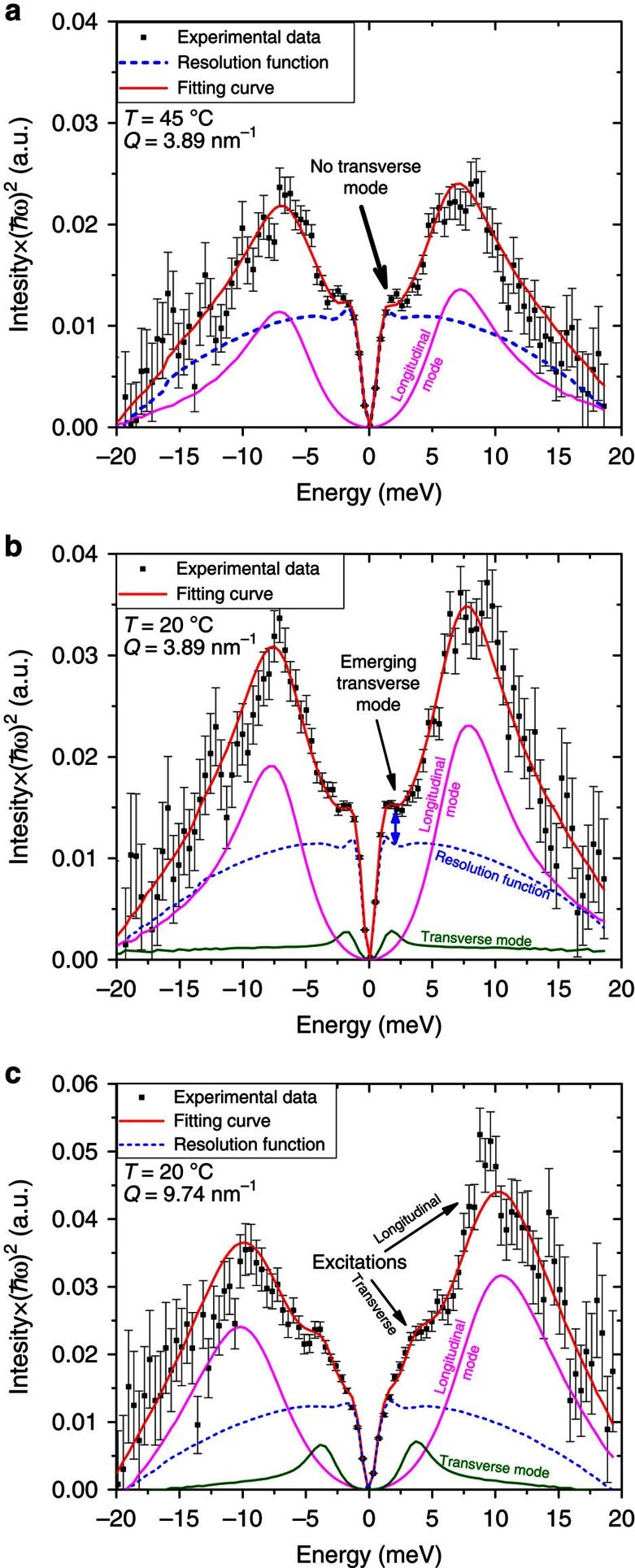
Propagating transverse excitations. Experimental data, the fitting curve and excitations from Fig. 2 for *Q*=3.89 nm^*−*1^ multiplied by (*ħω*)^2^ for 45 °C (**a**) and 20 °C (**b**), respectively. In **a**, the experimental data are well described by the sum of the RF and a single, longitudinal excitation only. In contrast, in **b**, the double blue arrow indicates excessive intensity in the experimental data above the RF+longitudinal mode, which can only be accounted for by the addition of the emerging transverse excitation with an inelastic shift below 2.5 meV. (**c**) *Q*=9.74 nm^*−*1^ for 20 °C: longitudinal modes dominate the intensity at *ħω ≈*10 meV and the propagating transverse excitation is clearly seen as a hump at *ħω≈*3.2 meV. The error bars represent 1 s.d.

An IXS spectrum from a liquid or disordered material is often dominated by the central elastic line and weaker inelastic excitations that appear as ‘shoulders'. Positive and negative energies correspond to the creation and annihilation of propagating modes, for example, phonon, respectively, whose intensities are governed by the detailed balance factor^39^. To extract the energy and the width of phonon excitations, experimental data were fitted utilizing a standard procedure^40^,^41^,^42^, that is, employing a model, which includes two inelastic terms, or modes, represented by two DHO profiles convoluted with the resolution function (RF) (see Methods section for details). Each DHO excitation has three varying parameters: *ω*(*Q*), *Γ*(*Q*) and *I*(*Q*), which represent the excitation energy, width and intensity, respectively. The other variables are the experimental background level and the intensity *S*(*Q*,0) of the central peak, whose measured shape coincides with the RF, which contains all spurious and geometrical factors affecting the measurement. The latter has been measured prior the current experiment and found to be close to the Voight function. Hence, in the fitting procedure the contribution of the central peak was described by varying a single parameter, that is, the amplitude *S*(*Q*,0), at each value of *Q (*see Fig. 1b). As a result, in the present experiment, the static structure along with processes related to slow molecular motions (for example, diffusion, relaxation and so on) unresolved with respect to the energy transfer are taken into account via a term proportional to *δ*(*ω*) and thus the measured central peak has an intensity *S*(*Q*,0), hereafter quoted as elastic intensity. As can be seen from Fig. 3, the IXS data cannot be fitted by only one excitation for the majority of the data. For example, at 20 °C, the IXS data cannot be fitted without the transverse excitation, which emerges at *ħω<*2.5 meV (see Fig. 3b); whereas at 45 °C (see Fig. 3a), the transverse excitation is heavily damped and the best fit is achieved just by summing the RF with the longitudinal excitation only. Although the main region of interest lays at *Q*<15 nm^−1^ revealing features of collective excitation dynamics, at *Q*-values above the peak of the structure factor the model with two DHOs also describes well the experimental data. One should note that at high *Q*-values excitations become heavily overdamped hence totally decaying within a single oscillation in time. Moreover, excitation frequencies and damping parameters acquire large error bars (see, below) indicating that the number of fitting parameters can be reduced, for example, via fixing *ω*(*Q*)=0, and thus assuming purely relaxational or diffusional motions. This, indeed, reduces error bars for the damping parameters. However, neither the expectation value nor *χ*^2^ would improve while simultaneously reducing fit quality. Therefore, we emphasize that the data cannot be satisfactorily described with a single Lorentzian function, but rather require at least two modes: either both of the non-propagating type (one purely relaxational and the other propagating with very large damping) or, alternatively, two overdamped modes. The latter model has the advantage that all data sets can be described in the same manner providing a direct comparison between fitting parameters at different temperatures over the whole range of *Q*.

**Figure 4:**
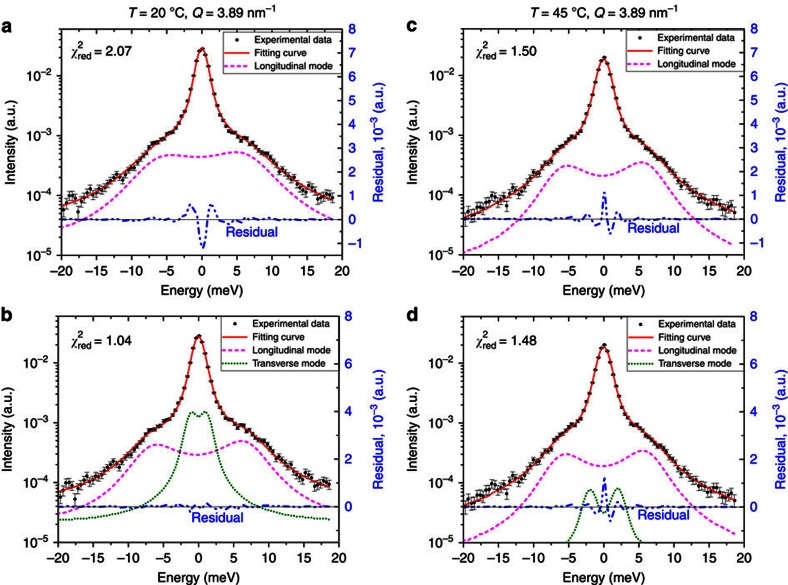
Statistical analysis of the IXS spectra at *Q*=3.89 nm^*−*1^. (**a**–**d**) The best model fits of the IXS scans at 20 °C (**a**,**b**) and 45 °C (**c**,**d**) at *Q*=3.89 nm^*−*1^ with one (longitudinal) and two (longitudinal and transverse) DHO excitations, respectively. The residuals are shown by blue dash-dotted curves. The resulting reduced χ^2^ is indicated in each panel. The error bars represent one s.d.

A more detailed statistical analysis of the IXS data at *Q*=3.89 nm^−1^ at both temperatures is presented in Fig. 4a–d. As can be seen from Fig. 4c,d, the data in fluid phase can be fitted with one DHO phonon branch only (longitudinal modes) and that the addition of the second branch does not improve the fit quality: the reduced χ^2^ change (from 1.50 to 1.48) is negligible had the second excitation been added to the model. Also, during the least squares fitting, irrespective of the starting parameters, the second DHO excitation tends to vanish, indicative of its inapplicability to the model. Conversely, in gel phase (Fig. 4a,b), the fit improves dramatically (the reduced χ^2^ changes from 2.07 to 1.04) when the second (transverse) excitation is added. Also, upon including a longitudinal excitation only (Fig. 4a), the residual curve exhibits the characteristic shape with two humps symmetrically located about zero energy transfer implying the deficiency of the second phonon excitation in the model.

**Figure 5:**
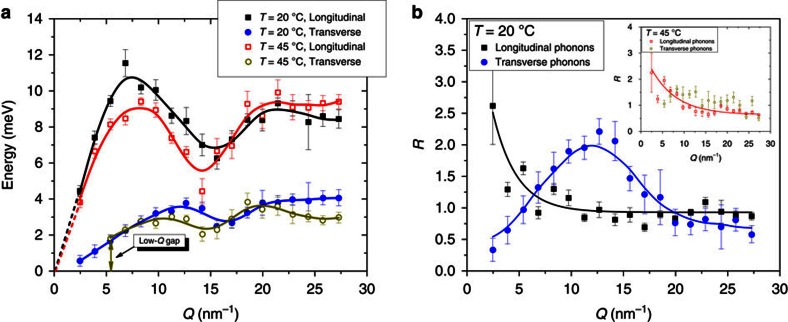
Phonon dispersion curves and damping ratios. (**a**) *ω*(*Q*) dispersion curves obtained using the DHO modelling. Filled and open symbols correspond to dispersions curves for 20 and 45 °C, respectively. Emergence of high-frequency transverse phonon gap at high temperature is indicated by the arrow. The solid lines are shown to guide eyes only. The dashed lines extrapolate the longitudinal phonon dispersions to *Q*=0. (**b**) The values of the damping ratio *R*=*ω*(*Q*)*/Γ*(*Q*) of the longitudinal and transverse branches are reported as a function of *Q*, at 20 °C. The solid lines through the symbols are guide to eyes. The inset displays *R* for the 45 °C. Since the *R* values for transverse phonons at 45 °C shows no specific trend (scattered near 1) the solid line was omitted. Errors bars represent 1 s.e.m.

The propagating nature of the observed transverse modes is unambiguously evidenced in Fig. 3c (at 20 °C, *Q*=9.74 nm^*−*1^) where a hump at *ħω≈*3.2 meV is clearly seen. The *ω*(*Q*) dispersion curves for both 20 and 45 °C measurements obtained as a result of the fitting procedures described above are presented in Fig. 5a. The DHO model fit to the IXS data revealed two phonon branches (longitudinal and transverse) both at low and high temperatures.

### Longitudinal phonons

In Fig. 5a, the behaviour of the longitudinal phonon dispersions is linear at low *Q* and its slope defines the high-frequency sound speed in the DPPC lipid for a given temperature. The speed of sound was estimated to be 2,532±190 m s^−1^ and 2,241±437 m s^−1^ for 20 and 45 °C, respectively. This result is in agreement with the molecular dynamics simulation of DPPC lipid^23^ and the previously reported^27^ values for a similar lipid, 1,2-dilauroyl-*sn*-glycero-3-phosphocholine (DLPC), measured by IXS. The obtained values of the speed of sound are lower than the high-frequency sound speed in bulk water at room temperature, which is 3,200 m s^−1^ (ref. 43). The minimum in the longitudinal dispersion, at *Q*_min_*≈*15.4 nm^*−*1^ for 20 °C and at *Q*_min_*≈*14.2 nm^*−*1^ for 45 °C, corresponds to the boundary of the quasi-Brillouin zone of a two-dimensional lipid, which can be understood to correspond to the mean distance *d* between the hydrocarbon chains of DPPC. This distance also corresponds to the first peak in the static structure factor *S*(*Q,0*) measured during the experiment (Fig. 1b). The *Q*_min_-values of the dispersion minimum (or the position of the chain correlation peak) provide a rough estimate of the areas per lipid molecule in both phases using a simplified equation:^44^ *A*_L_=2.64(9*d/*8)^2^, where *d*=2*π/Q*_min_, which yields *A*_L_=55.6*±*1 Å^2^ and 65.4*±*1 Å^2^ for 20 and 45 °C, respectively. These areas per molecule are in agreement with the ones obtained by coarse grained molecular dynamic simulation^45^ and more precise measurements^46^,^47^. Not unexpectedly, similar longitudinal phonon dispersions have been reported for deuterated 1,2-dimyristoyl-*sn*-glycero-3-phosphocholine (DMPC) using INS^30^. However, our results reveal that the minimum in the longitudinal dispersion relation is deeper in the fluid phase, which is supportive of previous IXS measurements^27^ but in contradiction with INS ones^30^. The inconsistency of the results measured by INS can possibly be attributed to the uncontemplated effects due to the selective deuteration of hydrocarbon tails used for the neutron scattering experiment.

### Transverse phonons

Sound propagates well in lipid membranes in the form of the longitudinal phonons described above. What is not obvious, though, is whether transverse phonons also occur in disordered systems such as lipid membranes. Because each phonon mode is responsible for the sound propagation and heat transfer, it is important to know how many modes occur in a lipid membrane of interest. In contrast to longitudinal phonons routinely observed in lipids, high-frequency transverse modes have not been experimentally observed in such systems so far. Recently, weakly dispersive transverse phonon modes were observed in DMPC membrane in the presence of ethanol. These modes were conferred as ‘out- of-plane', or propagating along the lipid tails, and were attributed to the influence of ethanol molecules on the bilayer^29^ since prior experimental and computational data for pure DMPC showed no sign of such transverse modes. In contrast, in this work we clearly observe high-frequency *in-plane* propagating transverse phonon modes in DPPC lipid membrane, similar to that observed in water and other liquids^48^,^49^. These modes should be clearly distinguished from the ‘out-of-plane' modes described in ref. 29.

In Fig. 5a transverse phonon modes are shown for both 20 and 45 °C. Transverse phonons behave similarly to longitudinal modes, exhibiting insignificant softening upon crossing the DPPC phase transition with a corresponding shift of *Q*_min_-value of the dispersion minimum to a smaller *Q*, revealing the increase of the mean distance *d* between hydrocarbon chains of DPPC in the L_α_ phase. The speed of sound estimated is 582±12 m s^−1^ for 20 °C and does not change noticeably within the experimental errors with the temperature rise. Recently, MD simulations of the thermal vibrations in DMPC lipid revealed surprisingly rich landscape of phonon excitations, which includes a longitudinal and a transverse acoustic modes, three optical branches with energies exceeding 10 meV, and an optical-like localized non-dispersive mode at about 6 meV (ref. 50). The good agreement between the longitudinal and the transverse excitations obtained by MD simulation^50^ and in our work suggests that previous oversimplified interpretation of the IXS spectra may not be appropriate.

Remarkably, at the high temperature, we also observe the low-*Q* phononic band gap, which can be identified as the disappearance of the transverse phonons at *Q<*5 nm^*−*1^. The phonon gap is indicated by the double arrow in Fig. 5a. This phenomenon can be explained in the framework of the recently developed phonon theory of liquids^36^,^37^,^38^, which predicts that transverse phononic gaps appear in disordered materials with increasing temperature. The first experimental evidence for such phononic gaps using IXS along with the theoretical explanation supported by MD simulations was shown for liquid Ar^48^. As can be seen further, the appearance of the phononic band gaps leads to important implications for membrane dynamics and passive membrane transport.

**Figure 6:**
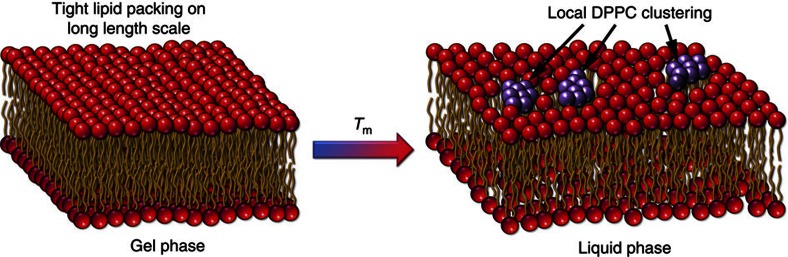
Formation of local DPPC clusters and pores. The schematic representation of the lipid bilayer transition from the L_β'_ to L_α_ phase. In the L_β'_ phase the lipids are tightly packed and thus the transverse phonons are supported over large distances. When the temperature is increased beyond *T*_m_ and the lipid bilayer undergoes the phase transition to L_α_ phase, the molecular arrangement is mostly disordered. However, due to thermal fluctuations, the local short-lived (on the order of a picosecond) lipid clustering with a size of ∼1.1–1.6 nm (or of the order of several lipids across) is triggered. The lipids, participating in the formation of these nanometre-sized clusters are schematically indicated by dark purple colour. The rarified regions around the dark purple DPPC clusters indicate the formation of transient pores on longer length scales.

As shown before^36^,^37^,^38^,^48^, the emergence of the phononic gaps is associated with diffusion and relaxation processes occurring in the lipid membrane. This point is of particular relevance, since the emergence of low-frequency phononic gaps (see Fig. 5a) implies that low-frequency wave-packets (long-wavelength limit) cannot propagate in a hybrid system (lipid membrane/water) due to the long-range structural disorder. In turn, the loss of long-range pair correlations activates diffusion processes at higher rates. This reveals two intrinsically different mechanisms of sound propagation and heat transfer in lipid membranes. The first mechanism is characterized by the short and intermediate length scales and is governed primarily by phononic excitations. The other mechanism is originated from highly diffusive dynamics due to heavily damped phonons at long-length scales. The cutoff value *Q*_gap_ at which the phononic gap occurs is related to the characteristic length-scale of a local ‘order', or clustering, beyond which the long-range propagation of phonons is impaired. Such local ‘clustering' can be attributed to the partial chain ordering in the lipid bilayer in a planar direction which is also characterized by the increased surface density of lipid molecules, the signature of the L_β'_-like arrangement (see Fig. 6). From the transverse phonon branch at 45 °C (see Fig. 5a) one can estimate that the characteristic size of a local ordering, or a cluster, *d*_gap_=2*π/Q*_gap_ is ∼1.1–1.6 nm, or of the order of several lipids across. Beyond *d*_gap_ the lipid molecules arrangement is disordered and does not support the in-plane transverse sound propagation, which is consistent with the L_α_ phase. Importantly, the observed clustering cannot be considered as a coexistence of the L_α_ and L_β'_ phases: since the IXS measures ultra-fast lipid dynamics (on the picosecond time scale), such local ordering should be considered as short time phonon-triggered density fluctuations. Consistently, low-*Q* phononic gaps were not observed in the L_β'_-DPPC, where the packing of the lipid acyl chains is much tighter and transverse phonon propagating mode is supported throughout measured *Q*-range. Such picosecond clustering was previously observed in liquid Sn and Ga^51^,^52^ and ascribed to transverse phonon localization on nanometre-length scales. Specifically, the cluster size was estimated 0.4–0.5 nm and 0.8–1.0 nm for Ga and Sn, respectively^51^,^52^.

To gain a deeper insight into the origin of the acoustic modes propagating throughout the membrane, in Fig. 5b we report the damping ratio *R*=*ω*(*Q*)*/Γ*(*Q*) (dimensionless value) at both temperatures. It is worth recalling that the condition *R*=1 marks the crossover between the overdamped (*R<*1) and underdamped (*R>*1) regime of acoustic propagation. In the former regime, the density fluctuation is so strongly damped that it dies-off before experiencing a single oscillation and thus the mode essentially acquires a diffusive, rather than oscillatory, character. On a more formal ground, one may recognize that in the strongly overdamped regime (*R*«1) the mode can be well described by a real-valued (merely dissipative) eigenvalue, while in the underdamped regime an imaginary (oscillatory) part emerges. The oscillatory character of the transverse perturbation is provided by shear restoring forces that in a liquid are weaker and much shorter lasting than in solids. Generally, *R* is a measure of the number of oscillations exhibited by the vibrational mode before it becomes damped off. A damped acoustic wave is described by a function, which has the general form:


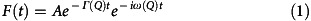


with the eigenvalue *Γ*(*Q*)*−iω*(*Q*) being complex, where *A* is a complex constant, *t* is the time, *Γ*(*Q*) is the damping coefficient, which determines the damping amplitude and *ω*(*Q*) is the oscillatory frequency. For example, in a perfect crystal, *Γ*(*Q*)*→*0 and the acoustic sound waves are long-lived, so *R→∞*. In this respect, the comparison shown in Fig. 5b is surprising. In particular, one readily notices that, at lower temperature, the transverse modes become increasingly solid-like at intermediate *Q* range where *R* reaches a clear maximum. At the same time, the longitudinal modes become more damped, reflecting the increasing difficulty of the system in supporting the high wavevector acoustic sound wave. In this *Q*-range, shear restoring forces cause the transverse waves to experience, within their lifetime, a number of oscillations even larger than the longitudinal modes. The situation is completely reversed in the L_α_ phase, where not only damping effects cause both transverse and longitudinal modes to become critically damped (*R≈*1) at high *Q*, but a clear gap prevents the shear mode from propagating over a large distance (see dispersion curve in Fig. 5a).

Our observation of the phononic gaps sheds light on the mechanism of the transmembrane solute diffusion. There has been much experimental evidence that the increased lipid packing reduces the diffusion across the membrane. For example, reducing the temperature across the lipid main transition or adding cholesterol leads to increased surface density and decreases the solute partitioning into the membrane^53^. Different explanations of the solute diffusion were proposed based on MD simulations. One model by Lieb and Stein proposed that solutes diffuse by ultra-fast ‘hopping', or ‘rattling' from void to void within the acyl chain region^54^,^55^. Another suggestion was that the partition coefficient strongly depends on the local chain ordering within the bilayer, which leads to the solute exclusion within the region of highly ordered chain packing^53^,^56^. Our experimental observations suggest that in the L_α_ phase, the thermally triggered density fluctuations promote short-time localization of the regions, or clusters, with highly ordered lipid chains within the membrane (similar to the L_β'_ phase), which in turn directly mediates the diffusion at longer length scales due to heavily damped phonons. Such local chain packing is short-living (on the picosecond time scale) and is localized on the scale of *d*_gap_, where the concentration of a solute can be potentially reduced. On the other hand, long-range disorder, where the transverse phonon propagation is impaired, can be a signature of short-lived volume voids (notice the rarefied lipid regions surrounding the DPPC clusters in Fig. 6) that facilitate the diffusion of small solute molecules *via* ‘hops' from one free volume void to another^56^.

The phononic landscape of biomembranes appears to be very rich^50^ and is still largely unexplored experimentally. The precise experimental determination of each phonon branch (be it acoustic or optical) using IXS remains very challenging for various reasons. For example, in our work, the study was done with an energy resolution of ∼1.7 meV, which, in combination with the Pseudo-Voigt line shape of the RF, hampered a more quantitative detection of low energy propagating transverse modes. Also, a relatively low *Q*-resolution (∼1.5 nm^−1^) makes it hard to determine the parameters (*Q*_gap_, *ω*_gap_) of the phononic gaps precisely. The development of new IXS spectrometers with higher energy and *Q* resolutions with sharper resolution line shape^57^ therefore, offers great opportunities to improve our understanding of the collective dynamics and the role it plays in the passive transport and other biological functions of biomembranes.

## Discussion

Here, we reveal for the first time, the existence of propagating transverse phonon modes in DPPC lipid membrane. Remarkably, we observe phononic gaps in the transverse phonon dispersion and evidence gap opening with increasing temperature. This result confirms the theoretical prediction^38^ that transverse phononic gaps appear in disordered materials over heat production due to the phonons interaction. Such band gaps are related to the diffusion and relaxation processes occurring in the lipid membrane. According to the phonon theory of liquids^36^,^37^,^38^, the band gap emerges when the transverse phonon propagation is no longer supported due to the increasing lipid chain disorder on long-length scales. For short and intermediate length scales, the band gap is an evidence of short-lived (on the picosecond time scale) local lipid clustering in lipid membranes. Such clustering (local chain ordering) supports the hypothesis that the local chain ordering may play an important role in solute diffusion by mediating the entropic expulsion (exclusion) of the solute from the inter-chain regions of the lipid membrane^53^. On the other hand, lipid chain disorder on long-length scale can potentially be an evidence for short-lived volume voids. Thermally triggered void formation can account for the abnormally fast diffusion of small solutes *via* hopping between voids^54^. The universal phonon-triggered mechanism may possibly help shed light on other permeation properties, such as the anomalously high proton permeability of lipid bilayers, which cannot be explained just in the framework of solubility-diffusion mechanism^58^. The formation of water wires, or fingers inside the short-lived voids (or transient defects) that are mediated by phonons and the heat transfer in the lipid bilayer can account for proton translocation along such hydrogen-bonded water chains.

## Methods

### DPPC lipid preparation and deposition

DPPC was purchased from Avanti Polar Lipids (Alabaster, AL) and used without further purification. An amount of 20 mg ml^−1^ stock solution of DPPC was prepared in a mixture of 3:2 hexane/2-propanol. Single crystal synthetic diamond substrate (4.5 *×* 4.5 mm^2^, 0.5 mm thick, (100) face orientation, purchased from Element Six Ltd. Ascot, UK) was cleaned with the consecutive rinses of chloroform, methanol and deionized water and then UV/ozone etched for 20 min before lipids deposition. DPPC solution was spin coated on the diamond substrate and spun for 60 s at 300 r.p.m., subsequently the speed was increased to 600 r.p.m. for another 60 s. The rotational frequency had been chosen such that no tossing of lipid solution from the substrate was observed^59^. The sample was dried subsequently in a vacuum oven at room temperature for 18 h to remove the remaining traces of solvent. As a result, well oriented DPPC multilayer containing up to a thousand bilayers was formed. The sample was measured at two temperatures: 20 °C (L_β'_ phase) and 45 °C (L_α_ phase) at the relative humidity of 97–98%. It should be noted, that a lipid sample prepared by the spin coating may exhibit imperfections, which in turn may affect the phonon spectra of disordered materials, like lipid membranes. Nevertheless, this can be easily observed by analyzing the phonon branches. For example, large clusters, aggregates or other forms of defects at micron-scale or larger (which are expected due to the spin-coating procedure) can be observed on the phonon spectra as absence of phonon excitations within the *Q*-range equal or less than 2*π/Q*, which is of the order of 10^*−*3^–10^*−*4^ nm^*−*1^, far below the resolution of any IXS spectrometer. Therefore, such defects cannot be seen in our experiment and they have no effect on the results presented in this work.

### Inelastic X-ray scattering experiment

The IXS scattering experiment was performed at the ID28 beamline at the European Synchrotron Radiation Facility, Grenoble, France. The spectrometer was operated at 21.747 keV incident energy, providing an energy resolution having a Pseudo-Voigt profile with full-width at half-maximum ∼1.7 meV and a beam focus of ∼250 × 80 μm^2^ (H × V, full-width at half-maximum). Further details of the experimental setup can be found elsewhere^40^. The DPPC multilayer sample was placed inside a humidity chamber where the sample temperature and the relative humidity can be precisely controlled. The scattering geometry used during the experiment is shown in Fig. 1a. X-ray beam impinges on the DPPC-covered diamond substrate at a very shallow angle (∼3 mrad) in order to optimize the scattering volume. The advantage of using a single-crystal diamond substrate is the absence of phonon-related scattering in the (*Q, ħω*) region of interest due to the very high sound velocities of diamond, as well as the absence of significant absorption. The scattering intensity is collected in the plane, parallel to the sample surface. Under these conditions, the scattering vector *Q* remains essentially within the DPPC lipid multilayer plane and, thus, only in-plane dynamics is probed. In this experiment a very wide *Q* range from 2.47 to 27.29 nm^*−*1^ was covered. A single IXS scan included 97 energy points with an exposure time of 90 s per point. The total data acquisition time for a given temperature (shown in Fig. 2) was ∼34 h. To cover *Q* range indicated above, 18 *Q*-values were measured at two different angles of the IXS spectrometer's detector arm. During the experiment, in order to mitigate the beam damage the sample was continuously scanned across the beam. Possible beam damage was examined both visually (by looking at the sample under a microscope) and by comparing the IXS scans for the same crystal analyser at the beginning and at the end of the experiment.

**Figure 7:**
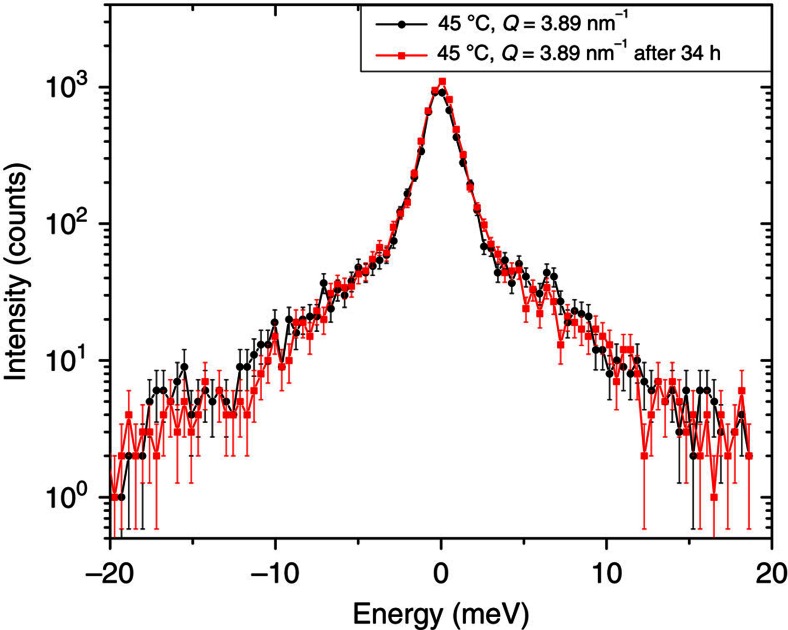
Assessment of the X-ray beam damage of DPPC. The comparison of the raw IXS scans (*T*=45 °C, *Q*=3.89 nm^−1^) at the beginning and at the end of the measurement with the total duration of the sample's exposure to the X-ray beam of ∼34 h. The error bars represent 1 s.d.

In Fig. 7, one can see the comparison of the raw IXS scans at 45 °C for *Q*=3.89 nm^−3^ at the beginning of the measurement and at the end. The total duration of the sample's exposure to the X-ray beam was ∼34 h. Neither significant deterioration of the sample nor the degradation of the IXS spectra quality was observed.

### Data availability

The data that support the findings of this study are available from the corresponding author upon request.

## Additional information

**How to cite this article:** Zhernenkov, M. *et al*. Revealing the mechanism of passive transport in lipid bilayers via phonon-mediated nanometer-scale density fluctuations. *Nat. Commun.* 7:11575 doi: 10.1038/ncomms11575 (2016).
