# Erratum: Revealing the mechanism of passive transport in lipid bilayers via phonon-mediated nanometre-scale density fluctuations

**DOI:** 10.1038/ncomms12292

**Published:** 2016-08-09

**Authors:** Mikhail Zhernenkov, Dima Bolmatov, Dmitry Soloviov, Kirill Zhernenkov, Boris P. Toperverg, Alessandro Cunsolo, Alexey Bosak, Yong Q. Cai

Nature Communications 7: Article number: 1157510.1038/ncomms11575 (2016); Published: 05 12 2016; Updated: 08 09 2016

This Article was originally published with an incorrect publication date. This paper was due to be published on 26 May 2016 together with other content on a similar topic, but due to an error was published earlier on the 12 May 2016. The publication date in both the PDF and HTML versions of the paper has been updated to reflect this.

